# 362. Saliva as a Reliable Sample Type for Mass SARS-CoV-2 Testing Strategies

**DOI:** 10.1093/ofid/ofab466.563

**Published:** 2021-12-04

**Authors:** Anne Wyllie, Chantal B Vogels, Orchid M Allicock, Anne Watkins, Mary Petrone, Devyn Yolda-Carr, Christina Harden, Doug Brackney, Chaney C Kalinich, Mallery I Breban, Isabel M Ott, Robby Sikka, Lolahon Kadiri, Nathan D Grubaugh

**Affiliations:** 1 Yale School of Medicine, New Haven, Connecticut; 2 Yale School of Public Health, New Haven, Connecticut; 3 Minnesota Timberwolves, Minneapolis, Minnesota; 4 Yale University, New Haven, Connecticut

## Abstract

**Background:**

Quickly detecting and isolating individuals positive for SARS-CoV-2 is essential for limiting virus spread. Policy makers rely on the number of active cases to make decisions, and individuals use this information to evaluate risk should they return to public spaces. Robust testing strategies have been plagued with limited authorized diagnostic assays and high test prices, with large-scale implementation hampered by worldwide supply chain issues.

**Methods:**

Having identified its potential early in the pandemic, we simplified saliva-based COVID-19 diagnostic testing by (1) not requiring collection tubes with preservatives, (2) replacing nucleic acid extraction with a simple enzymatic and heating step, and (3) testing specimens for SARS-CoV-2 in dualplex RT-qPCR. Moreover, we validated this approach (“SalivaDirect”) with reagents and instruments from multiple vendors to circumvent supply chain disruptions.

**Results:**

SalivaDirect’s simplified protocol does not compromise on sensitivity. In our hospital cohort, we found a high positive agreement (94%) between saliva tested with SalivaDirect and nasopharyngeal swabs tested with a commercial RT-qPCR kit. With the National Basketball Association we tested 3,779 saliva specimens from healthy individuals and detected low rates of invalid (0.3%) and false-positive (< 0.05%) results. Using comparative assays and sample types, we also demonstrated SalivaDirect to efficiently detect SARS-CoV-2 in asymptomatic individuals.

SalivaDirect is a simplified method for SARS-CoV-2 detection

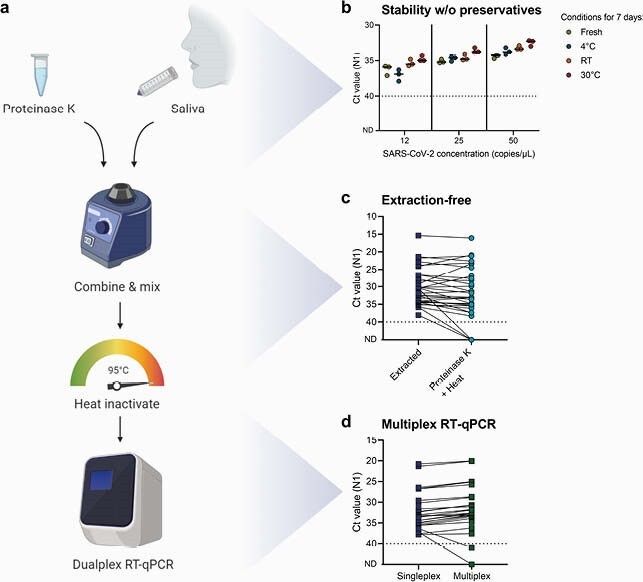

(A) Schematic overview of SalivaDirect workflow depicting the main steps of mixing saliva with proteinase K, heat inactivation, and dualplex qRT-PCR testing. Figure created with Biorender.com. (B) SARS-CoV-2 is stable in saliva for at least 7 days at 4C, room temperature (RT; 19C), and 30C without addition of stabilizing buffers. Spiked-in saliva samples of low virus concentrations (12, 25, and 50 SARS-CoV-2 copies/mL) were kept at the indicated temperature for 7 days and then tested with SalivaDirect. N1 cycle threshold (Ct) values were lower when kept for 7 days at 30C as compared to fresh specimens (Kruskal-Wallis; p = 0.03). Horizontal bars indicate the median. (C) Comparing Ct values for saliva treated with proteinase K and heat as compared to nucleic extraction yields higher N1 Ct values without extraction (Wilcoxon; p < 0.01). (D) Testing extracted nucleic acid from saliva with the N1 primer-probe set (singleplex) as compared to a multiplex assay showed stronger N1 detection in multiplex (Wilcoxon; p < 0.01). The dotted line in (B)–(D) indicates the limit of detection.

**Conclusion:**

Saliva is a valid alternative to swabs for SARS-CoV-2 screening. Importantly, SalivaDirect enables labs to utilize existing infrastructure, improving test implementation time and requiring limited investment to scale-up to meet mass testing needs. With the safe and reliable self-collection of saliva, our vision is to help provide accessible and equitable testing solutions, especially in low-resource and remote settings.

**Disclosures:**

**Anne Wyllie, PhD**, **Global Diagnostic Systems** (Consultant)**Pfizer** (Advisor or Review Panel member, Research Grant or Support)**PPS Health** (Consultant)**Tempus Labs, Inc** (Research Grant or Support) **Nathan D. Grubaugh, PhD**, **Tempus Labs** (Consultant)

